# E4bp4-Cyp3a11 axis in high-fat diet-induced obese mice with weight fluctuation

**DOI:** 10.1186/s12986-024-00803-1

**Published:** 2024-05-27

**Authors:** Shuoshuo Sun, Ruixiang Zhang, Yu Chen, Yijiao Xu, Xingjia Li, Chao Liu, Guofang Chen, Xiao Wei

**Affiliations:** 1 Department of Endocrinology, Affiliated Hospital of Integrated Chinese and Western Medicine, Nanjing University of Chinese Medicine, Nanjing, 210028 People’s Republic of China; 2 Jiangsu Province Academy of Traditional Chinese Medicine, Nanjing, 210028 People’s Republic of China; 3 The Second Affiliated Hospital of Wenzhou Medical University, Wenzhou, 325000 People’s Republic of China

**Keywords:** Obesity, High-fat diet, Liver, Cyp3a11, E4bp4

## Abstract

**Objective:**

Weight regain after weight loss is a challenge in obesity management. The metabolic changes and underlying mechanisms in obese people with weight fluctuation remain to be elucidated. In the present study, we aimed to profile the features and clinical significance of liver transcriptome in obese mice with weight regain after weight loss.

**Methods:**

The male C57BL/6J mice were fed with standard chow diet or high-fat diet (HFD). After 9 weeks, the HFD-induced obese mice were randomly divided into weight gain (WG), weight loss (WL) and weight regain (WR) group. After 10 weeks of dietary intervention, body weight, fasting blood glucose (FBG), intraperitoneal glucose tolerance, triglycerides (TG), total cholesterol (T-CHO) and low-density lipoprotein cholesterol (LDL-C) were measured. Morphological structure and lipid droplet accumulation in the liver were observed by H&E staining and oil red O staining, respectively. The liver transcriptome was detected by RNA sequencing. Protein expressions of liver cytochrome P450 3a11 (Cyp3a11) and E4 promoter-binding protein 4 (E4bp4) were determined by Western blot.

**Results:**

After 10 weeks of dietary intervention, the body weight, FBG, glucose area under the curve, T-CHO and LDL-C in WL group were significantly lower than those in WG group (*P* < 0.05). At 4 weeks of HFD re-feeding, the mice in WR group presented body weight and T-CHO significantly lower than those in WG group, whereas higher than those in WL group (*P* < 0.05). Hepatic vacuolar degeneration and lipid droplet accumulation in the liver were significantly alleviated in WL group and WR group, compared to those in WG group. The liver transcriptome associated with lipid metabolism was significantly altered during weight fluctuation in obese mice. Compared with those in WG group, Cyp3a11 in the liver was significantly upregulated, and E4bp4 was significantly downregulated in WL and WR groups.

**Conclusion:**

Obese mice experience weight regain after weight loss by HFD re-feeding, but their glucose and lipid metabolism disorders are milder than those induced by the persistence of obesity. Downregulated E4bp4 and upregulated Cyp3a11 are detected in obese mice after weight loss, suggesting that the E4bp4-Cyp3a11 axis may involved in metabolic mechanisms underlying weight regulation.

**Graphical Abstract:**

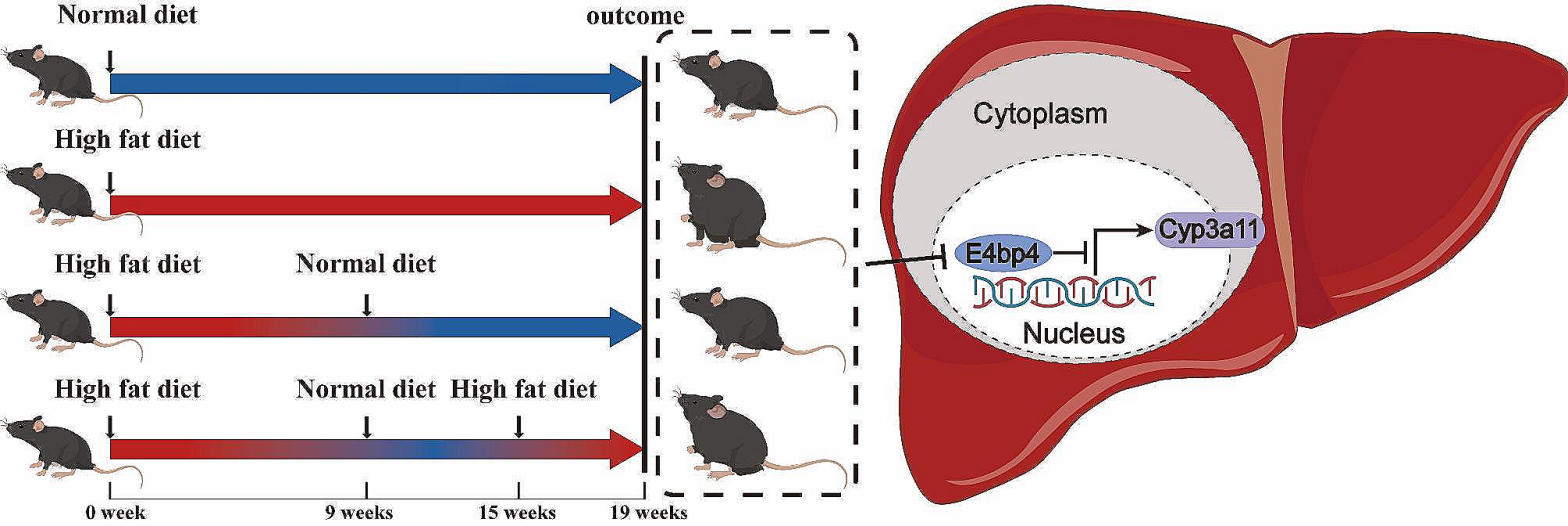

## Introduction

Obesity, a chronic metabolic disease characterized by an excessive accumulation of body fat, has an ever-rising prevalence worldwide, with the overweight or obese people accounting for one-third of the global population [1–4]. As a potential risk factor for many chronic diseases, obesity increases susceptibilities to type 2 diabetes mellitus (T2DM), nonalcoholic fatty liver disease (NAFLD), stroke, myocardial infarction, hypertension and tumors, which significantly reduces the quality of life and life expectancy [4–6]. Effective approaches to prevent and control obesity are urgently needed. Currently, lifestyle interventions (mainly diet and exercise), medication and bariatric surgery are major therapeutic strategies for obesity. However, the limited types and adverse events of weight-loss drugs, and the invasiveness and strict indications of bariatric surgery are great obstacles in treating obesity [7–9]. Dietary intervention combined with exercise is the most prevalent method, but often challenged by a poor compliance.

The effect of weight loss from dietary intervention can be just maintained in a low proportion of obese people. Generally, over 80% of them experience weight regain after weight loss within one year [10–12]. It is found that weight regain after weight loss is an independent risk factor for stroke, diabetes and coronary heart disease [13]. Compared with the persistence of obesity, weight regain after weight loss in mice aggravates obesity-associated metabolic disorders in the liver [13, 14]. On the contrary, experimental data also support more metabolic benefits provided by weight regain after weight loss, compared to the persistence of obesity [15, 16]. Sougiannis et al. [15] have reported that obese mice experiencing a slight weight regain after weight loss (high-fat diet [HFD] switches to low-fat diet [LFD] and then re-switches to HFD) exhibit a lower degree of hepatic steatosis, compared with that of obese mice fed with HFD for a long period. Whether weight rebound is favorable to metabolism remains controversial. In the present study, we compared the effects of weight regain after weight loss versus persistent obesity on liver metabolism in mice.

The liver serves to maintain lipid homeostasis by mediating lipid uptake, synthesis and catabolism [17, 18]. Excessive fat accumulation impairs liver function and further exacerbates obesity-associated glucose and lipid metabolism disorder in obese people [19, 20], including dyslipidemia, hepatic steatosis, insulin resistance, and T2DM [17, 21]. Therefore, an important link has been proposed to exist between hepatic lipid metabolism and obesity [22].

At present, there is neither a clear definition of weight rebound, nor a well-recognized in vivo weight rebound model [23]. The switch from HFD to normal diet and then HFD again is usually adopted to induce obesity, weight loss and weight rebound in mice. In the present study, using a similar induction method, we profiled the liver transcriptome and screened differentially expressed genes (DEGs) in mice between WG group, WL group and WR group. Our findings illustrated the significance of the E4bp4-Cyp3a11 axis in the weight management of obese mice.

## Methods and materials

### Animal experiments

A total of 40 male C57BL/6J mice aged 6–8 weeks were habituated at the specific pathogen free (SPF) level for 3 weeks in the Experimental Animal Center of the Affiliated Hospital of Integrated Chinese and Western Medicine Nanjing University of Chinese Medicine. The mice were fed with a standard chow diet (10% kcal from fat, set as the normal control group, *n* = 10) or a high-fat diet (HFD, D12492 rodent diet) composed of 60% fat and 20% carbohydrate (*n* = 30). After 9 weeks of feeding, the HFD-induced obese mice were randomly divided into three groups (*n* = 10 each) including weight gain (WG, fed with HFD for 10 weeks), weight loss (WL, fed with standard chow diet for 10 weeks), and weight regain (WR, standard chow diet for 6 weeks, followed by HFD for 4 weeks) groups. Mice were euthanized and sampled after different dietary interventions for 10 weeks. All mice were given free accesses to water, and housed in an environment at standard temperature and humidity in a 12 h/12 h light-dark cycle. Rodent HFD and ND were provided by Jiangsu Xietong Pharmaceutical Bio-engineering Co., Ltd. Animal experimental procedures were approved by the Ethics Committee of the Affiliated Hospital of Integrated Chinese and Western Medicine Nanjing University of Chinese Medicine (Ethics No. AEWC-20210511-142), and followed the *Guide for the Care and Use of Laboratory Animals*.

### Intraperitoneal glucose tolerance test (IPGTT)

After different dietary interventions for 10 weeks, IPGTT was performed at 3 days before sacrificing. Following a 16-h fasting without water deprivation, the mice were intraperitoneally injected with glucose (2 g/kg). Blood glucose concentrations in the venous were subsequently measured at 0, 30, 60, and 120 min after glucose injection, using the commercial glucose assay kit (Nanjing Jiancheng Bioengineering Institute).

### Sample collection and lipid profile measurement

The mice were anesthetized with intraperitoneal injection using pentobarbital sodium at a dose of 100 mg/kg. Liver tissues were harvested, fixed in 4% paraformaldehyde, dehydrated, and paraffin-embedded. Part of tissues were prepared into frozen sections; the remaining tissues were placed in liquid nitrogen and stored at -80 °C. Blood samples were collected through retro-orbital bleeding, centrifuged at 4 °C, 3000 r/min for 10 min, and the supernatant was collected into 1.5 ml centrifuge tubes, stored at -80 °C for further use.

Mouse triglycerides (TG), total cholesterol (T-CHO) and low-density lipoprotein cholesterol (LDL-C) were measured using commercial detection kits provided by Nanjing Jiancheng Bioengineering Institute. Paraffin-embedded sections and frozen sections of mouse liver tissues were sliced and stained with hematoxylin and eosin, and oil red O, respectively. Morphology and lipid droplet accumulation in mouse liver tissues were observed using the Olympus light microscope.

### RNA sequencing

RNA sequencing (RNA-seq) was performed on mouse liver samples in the Majorbio Biotech Co., Ltd. Briefly, mouse liver samples were processed by extraction of total RNA, isolation of mRNA using oligo(dT)-coated magnetic particles, mRNA fragmentation, reverse transcription to cDNA, adaptor ligation, screening of RNA fragments and preparation of a library using the NovaSeq 6000 system. DEGs were screened, and enriched signaling pathways were predicted using the Kyoto encyclopedia of genes and genomes (KEGG) Pathway Database.

### Western blot

Mouse liver tissues were lysed in RIPA Lysis Buffer (Beyotime Institute of Biotechnology), followed by centrifugation at 12,000 r/min for 30 min and preparation of protein samples. Protein concentration was measured using the BCA Protein Assay Kit (Beyotime). Protein samples were separated by SDS-PAGE, loaded on polyvinylidene fluoride (PVDF) membranes (Millipore), cut into small pieces according to the interested molecular size, immersed in 5% non-skimmed milk and immunoblotted with primary antibodies overnight at 4℃, including the anti-Cyp3a11 antibody (1:3000, Proteintech), anti-E4bp4 antibody (1:3000, Proteintech) and anti-β-actin antibody (1:3000, Cell Signaling Technology). On the next day, they were immunoblotted with secondary antibodies (1:5000, Cell Signaling Technology) for 1 h at room temperature. Bands were exposed using the ECL Western Blotting Substrate (Vazyme) and visualized using the Tanon 5200 Chemiluminescent Imaging System.

### Immunohistochemistry

Paraffin-embedded sections were prepared for deparaffinization, antigen retrieval, and incubation with primary and secondary antibodies for immunohistochemistry. After counter-staining dehydration, and mounting, the positive expression of Cyp3a11 and E4bp4 protein in liver tissues was observed under an optical microscope.

### Statistical analysis

Statistical analysis was performed using SPSS 22.0. Data were expressed as mean ± standard deviation (SD) and data of three or more groups were compared by one-way ANOVA. *P* < 0.05 was considered as statistically significant.

## Results

### Switch of HFD to ND alleviates glucose and lipid metabolism disorders in obese mice

#### Switch of HFD to ND lowers body weight and fasting blood glucose in obese mice

**Fig. 1:**
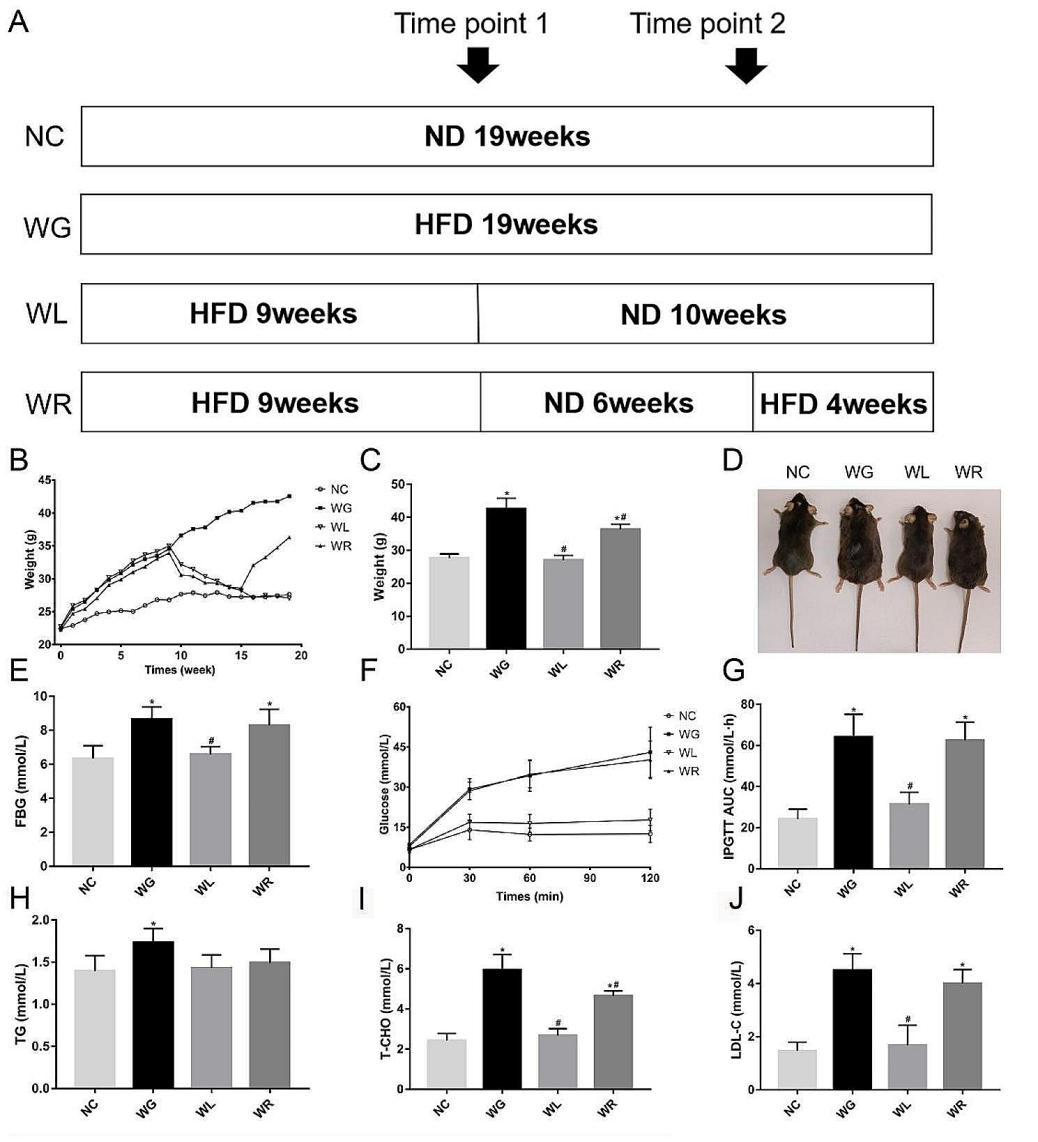
Switch of HFD to ND alleviates glucose and lipid metabolism disorder in obese mice. (**A**) Dietary interventions in each group. Mice in NC, WG, WL and WR groups were fed with ND for 19 weeks; HFD for 19 weeks; HFD for 9 weeks and ND for 10 weeks; and HFD for 9 weeks, ND for 6 weeks and HFD for 4 weeks, respectively. (**B**) Changes in the mouse body weight during the experimental period. (**C**) Mean body weight of mice at the end of the dietary intervention. (**D**) Representative macroscopic image of mice at the end of the dietary intervention. (**E**-**J**) FBG (**E**), glucose level at each time point after glucose injection (**F**), mean glucose area under the curve (**G**), mean TG level (**H**), the mean T-CHO level (**I**) and mean LDL-C level (**J**) in each group. *n* = 10. ^*^P < 0.05 vs. NC group; ^#^*P <* 0.05 vs. WG group

After 10 weeks of dietary intervention, the mean body weight and FBG in WG group were significantly higher than those in NC group (*P* < 0.05), but both indexes were significantly lower in WL group than in WG group (*P* < 0.05). The mean body weight was significantly lower in WR group than in WG group, but higher than in WL group (*P* < 0.05). No significant difference was detected in FBG between WR group and WG group (*P* = 0.286) (Fig. 1B-E).

#### Switch of HFD to ND improves glucose tolerance in obese mice

The blood glucose curve for IPGTT in WG group was compared with that in NC group. FBG and glucose levels at each time point after glucose injection were significantly higher in WG group (*P* < 0.05). Compared with those in WG group, the glucose area under the curve was reduced in WL group, with significantly lower FBG and glucose levels at each time point after glucose injection (*P* < 0.05). The glucose area under the curve was comparable between WR group and WG group (*P* > 0.05) (Fig. 1F, G).

#### Switch of HFD to ND lowers blood lipid levels in obese mice

TG, T-CHO and LDL-C levels were significantly higher in WG group than in NC group (*P* < 0.05). T-CHO and LDL-C levels in WL group were significantly lower than those in WG group (*P* < 0.05). T-CHO level was significantly lower in WR group than in WG group, but significantly higher than that in WL group (*P* < 0.05) (Fig. 1H-J).

### Switch of HFD to ND reduces lipid droplet accumulation in the liver of obese mice

**Fig. 2:**
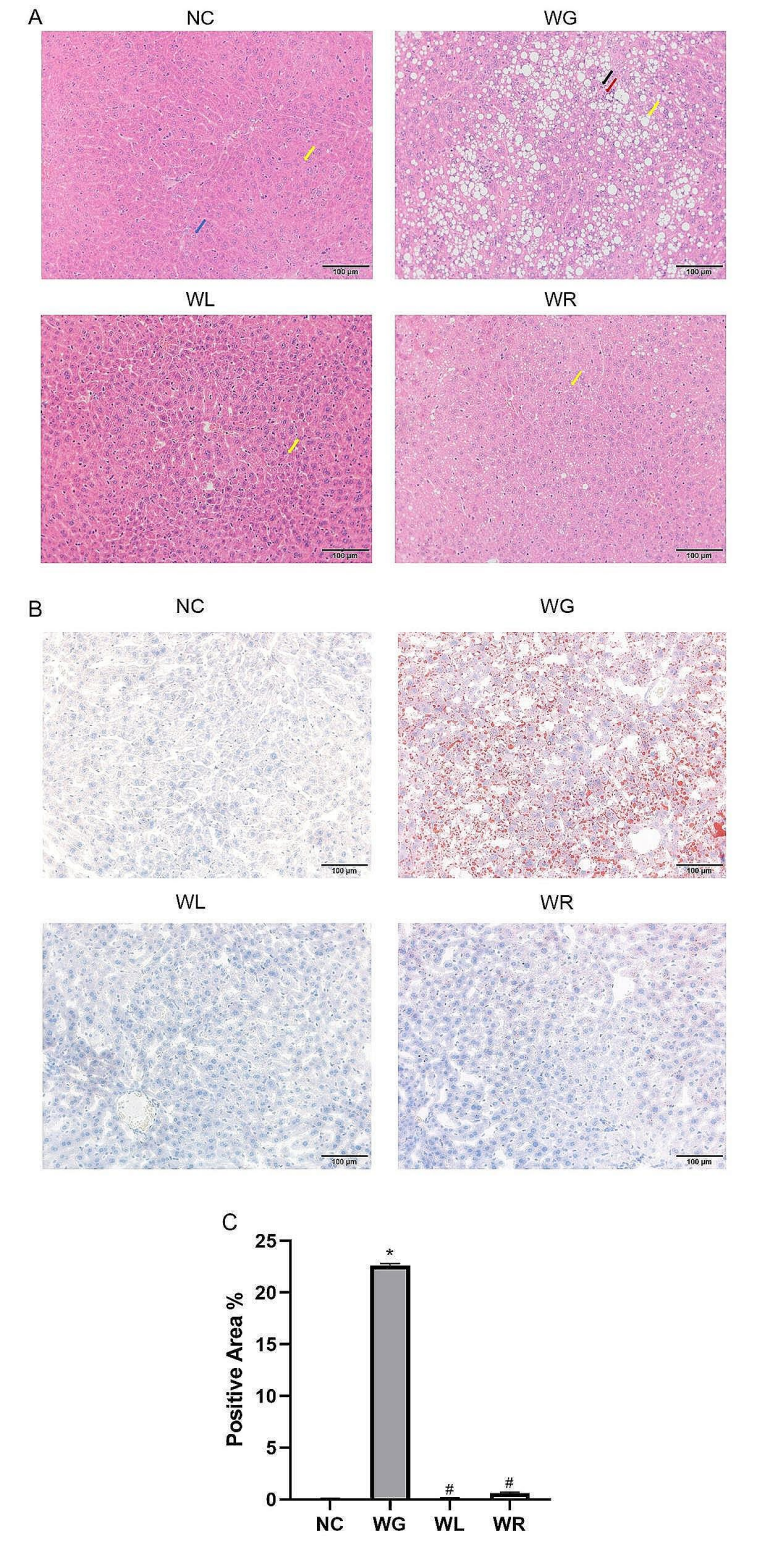
H&E staining and oil red O staining of mouse liver tissues in NC, WG, WL and WR groups, respectively. Blue arrows indicate hydropic degeneration of hepatic cell, yellow arrows indicate hepatic steatosis, black arrows indicate spotty necrosis, red arrows indicate pyknosis and karyorrhexis along with a small amount of granulocytic infiltration (**A**). Oil red O staining and related quantitative results, in this image the steatosis segments are red stained by oil red O and positive area were quantified with Aipathwell softwell (**B**, **C**). Magnification = 200×. ^*^P < 0.05 vs. NC group; ^#^*P <* 0.05 vs. WG group

Compared with those in NC group, vacuolar degeneration and lipid droplet accumulation in the liver were much more pronounced in WG group, but not observed in WL group. Compared with those in WG group, vacuolar degeneration and lipid droplet accumulation in the liver were significantly alleviated in WR group (Fig. 2A, B).

### Switch of HFD to ND alters the liver transcriptomic profile of obese mice

#### Gene expression profile in the mouse liver is similar among four groups

**Fig. 3:**
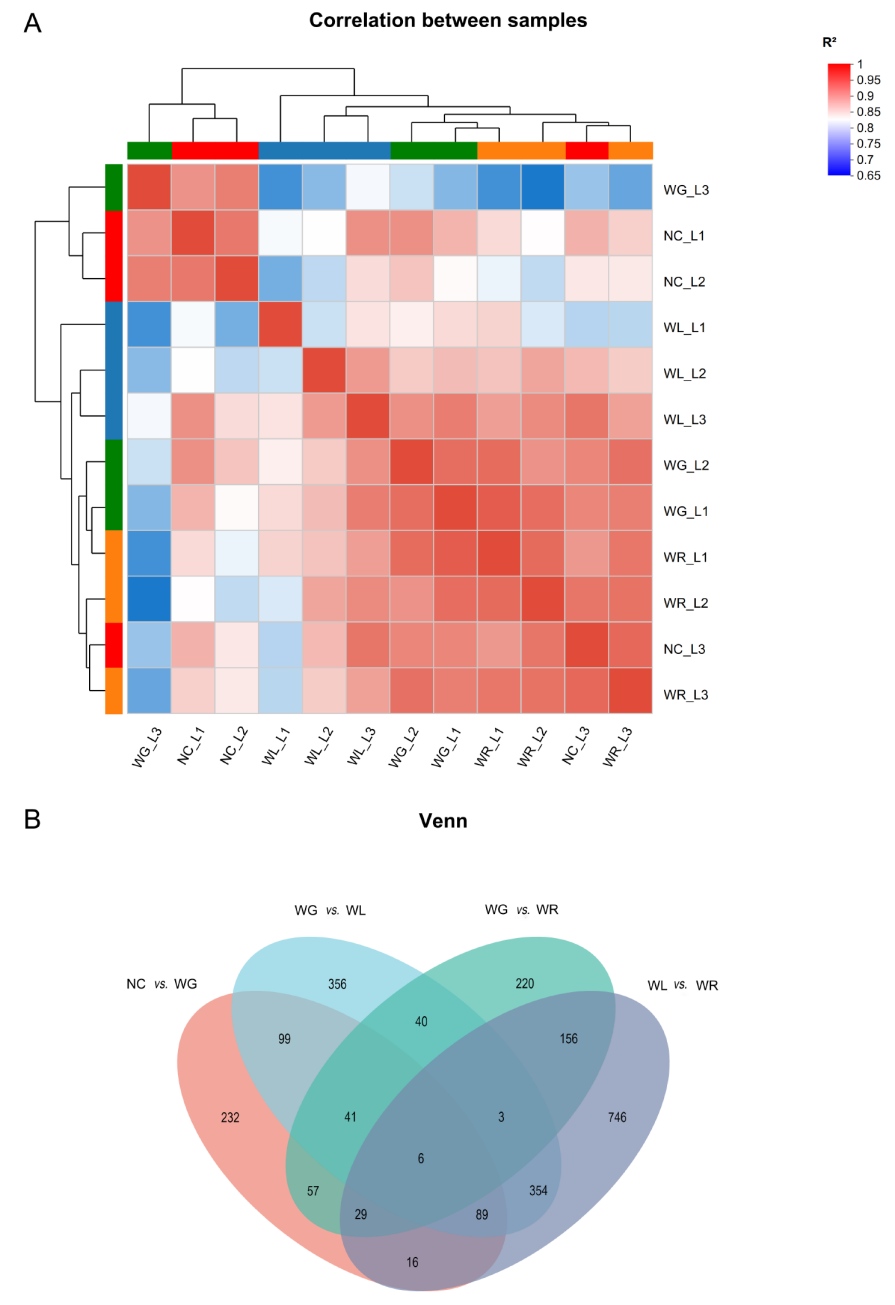
Heatmap of liver transcriptome profile (**A**) and Venn diagram of DEGs in the liver transcriptome (**B**). Three liver samples (L1, L2 and L3) were harvested in each group for analyzing the liver transcriptome

Gene expression profiles in liver samples were illustrated to identify the correlations of gene expression profiles among NC, WG, WL and WR group. As shown in the heatmap, the correlation coefficients (R^2^) were all above 0.75, and most of them were above 0.90. The proximity of R^2^ to 1 indicated that the gene expression profiles were highly similar among four groups, ensuring the reliability of the following findings (Fig. 3A).

#### DEGs in the liver transcriptome

DEGs with |log_2_fold change|≥1 and *P* < 0.05 were screened in the liver transcriptomes of NC, WG, WL and WR groups. We depicted a Venn diagram to illustrate the intersection of DEGs among the four groups (Fig. 3B). A total of 569 DEGs between NC group and WG group, 988 DEGs between WG group and WL group, 552 DEGs between WG group and WR group, and 1399 DEGs between WL group and WR group were identified. A total of 235 DEGs among NC, WG and WL groups, 133 DEGs among NC, WG and WR groups, and 90 DEGs among WG, WL and WR groups were identified. Finally, 47 DEGs were identified among NC, WG, WL and WR groups.

### Switch of HFD to ND dysregulates signaling pathways associated with lipid metabolism in the liver of obese mice

**Fig. 4:**
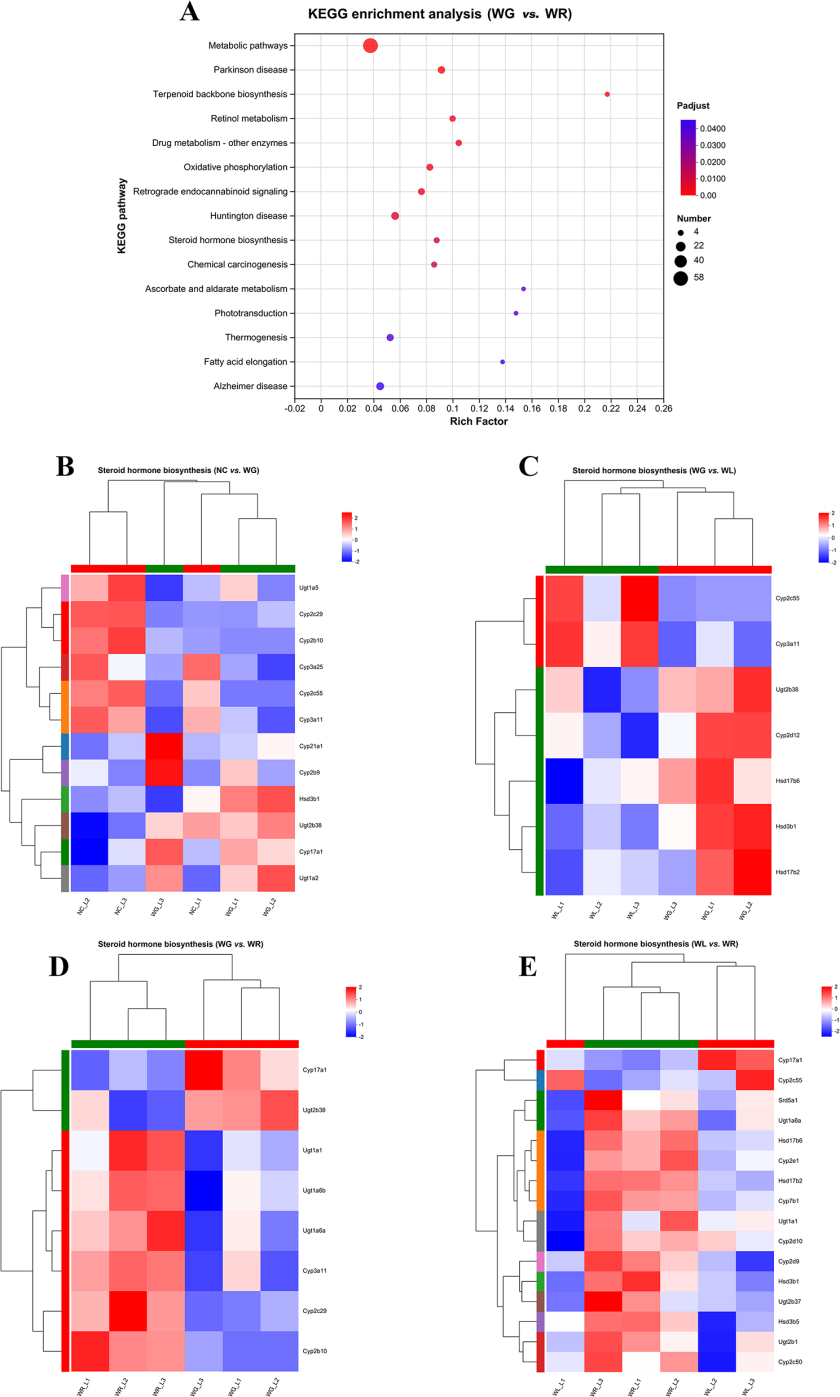
Switch of HFD to ND dysregulates signaling pathways associated with lipid metabolism in the liver of obese mice. (**A**) Bubble diagram of KEGG pathway enrichment analysis on DEGs between WG and WR groups. (**B**-**E**) DEGs enriched in the steroid hormone biosynthesis pathway between NC and WG groups (**B**), WG and WL groups (**C**), WG and WR groups (**D**), and WL and WR groups (**E**)

Signaling pathways associated with lipid metabolism and enriched in DEGs were predicted by KEGG. As shown in the bubble diagram of KEGG pathway enrichment analysis, steroid hormone biosynthesis, fluid shear stress, atherosclerosis and bile secretion pathways were mainly enriched in DEGs between WL group and WG group. Oxidative phosphorylation, steroid hormone biosynthesis and fatty acid elongation pathways were mainly enriched in DEGs between WG group and WR group (Fig. 4A). Interestingly, the steroid hormone biosynthesis pathway was enriched in DEGs between any two of the four groups. Cytochrome P450 3a11 (Cyp3a11) was significantly downregulated in WG group compared to NC group (Fig. 4B), and significantly upregulated in WL and WR groups compared to WG group (Fig. 4C, D). Its expression level was comparable between WL and WR groups (Fig. 4E).

### Switch of HFD to ND downregulates E4bp4 and upregulates Cyp3a11 in the liver of obese micev

**Fig. 5:**
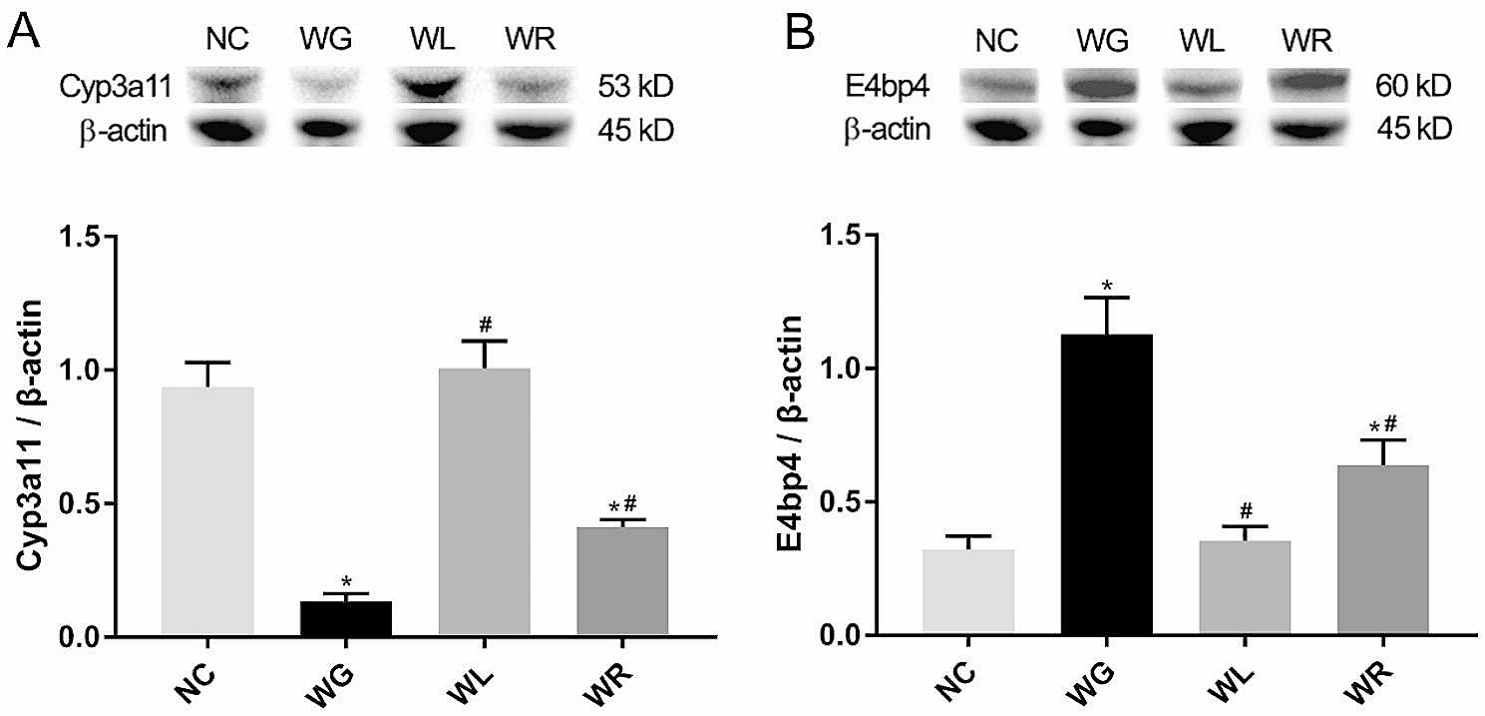
Switch of HFD to ND downregulates E4bp4 and upregulates Cyp3a11 in the liver of obese mice. Protein expressions of Cyp3a11 (**A**) and E4bp4 (**B**) in mouse liver. ^*^P < 0.05 vs. NC group; ^#^*P <* 0.05 vs. WG group

We detected the protein levels of Cyp3a11, and its upstream transcription factor E4bp4 in mouse liver tissues by both immunohistochemistry (Fig. 5A) and western blot (Fig. 5B). Cyp3a11 was significantly downregulated in WG group compared to NC group, but significantly upregulated in WL and WR groups compared to WG group (*P* < 0.05). The protein level of Cyp3a11 was consistent with its mRNA level identified in the liver transcriptome of obese mice. The protein expression of E4bp4 was significantly upregulated in WG group compared to NC group, but significantly downregulated in WL and WR groups compared to WG group (*P* < 0.05).

## Discussion

A long-term HFD induces obesity [24, 25], and causes lipid hypermetabolism in the liver [26]. In the present study, weight regain after weight loss in mice was induced by switching HFD to ND and then re-switching to HFD. We mainly analyzed the physiological and metabolic changes in mice, and the underlying mechanism. Transcriptomic analysis showed that lipid metabolism-related genes were differentially expressed in the liver during weight fluctuation of obese mice. We later found that the E4bp4-Cyp3a11 axis maybe responsible for weight regain after weight loss in obese mice.

Our data revealed that after switching HFD to ND, obese mice presented weight loss, and significant reduction in FBG, glucose area under the curve and blood lipids, and lipid droplet accumulation in the liver. After HFD re-feeding, obese mice experienced weight regain, although their body weight, blood lipids and lipid droplet accumulation in the liver were significantly lower than those in the mice continuously fed with HFD. A relevant study has consistently illustrated that the body weight of mice fed with low-fat diet for 7 weeks and then HFD for 4 weeks does not rebound to the level before weight loss, but remains lower than that of HFD-fed mice; moreover, their glucose and lipid metabolism are also more healthier than those in HFD-fed mice [15]. However, Kyung et al. [27] have reported that body weight, FBG and cholesterol re-elevate in mice fed with HFD for 8 weeks, followed by the feeding of ND for 4 weeks and HFD for another 4 weeks, suggesting the potential harm of weight rebound after weight loss. The controversial findings may be attributed to differences in dietary intervention and weight loss duration that influence the glucose and lipid metabolism and the degree of weight rebound.

The emergence of transcriptomics makes it easier to explore phenotypes of diseases. Through comparing transcriptomic differences between normal sates and pathological states, transcriptomics has been used to identify disease-related diagnostic or therapeutic genes or targets, to predict the function of the target genes, and infer the pathogenic mechanism and drug targets. Since liver is the core organ responsible for metabolism, the present study investigated the impacts of transcriptomic changes during weight fluctuations on glycolipid metabolism and hepatic lipid deposition, with the aim of providing new targets for the treatment of obesity, NAFLD, and other metabolic diseases. Here, the transcriptomic profile in the liver was analyzed during the process of weight rebound after weight loss. It is found that signaling pathways associated with lipid metabolism were enriched in a large number of DEGs between WG and WR group, including the steroid hormone biosynthesis pathway. We speculated that the liver transcriptome may be responsible for weight regain after weight loss via mediating lipid metabolism in mouse liver. RNA-seq further identified that Cyp3a11 was differentially expressed during weight fluctuation. Cyp3a11 (human homolog Cyp3a4) is a member of the cytochrome P450 (Cyp450) superfamily and widely present in mouse liver. Functionally, Cyp3a11 is involved in drug detoxification and lipid metabolism in the liver [28, 29].

Zeng et al. [30] have reported that the mRNA and protein levels of Cyp3a11 are downregulated in the liver of HFD-fed mice, palmitic acid-induced primary mice hepatocytes, palmitic acid-induced HepG2 cells, as well as the liver of NAFLD patients [31]. Consistently, our data also showed that the mRNA and protein levels of Cyp3a11 were significantly downregulated in the liver of HFD-fed mice compared with ND-fed mice. Compared with that of obese mice, the expression level of Cyp3a11 was significantly higher in the liver of obese mice experiencing weight regain after weight loss. Hence, we believed that Cyp3a11 is involved in liver transcriptomic change during weight fluctuation induced by dietary interventions.

The correlation of Cyp3a11 with weight fluctuation in obese mice may be mediated by its upstream transcription factors. In the present study, E4bp4 was identified as a DEG among the four groups. E4bp4, also known as nuclear factor interleukin 3-regulated (NFIL3), is a member of the basic leucine zipper (bZIP) family [32]. It is widely distributed in the liver, serving as a vital regulator for lipid metabolism [33]. Inhibiting the activation of E4bp4 in the liver has been demonstrated as a potential therapeutic target for hepatic steatosis [33, 34]. In high-fat, low-methionine, and choline-deficient (HFLMCD) diet-induced nonalcoholic steatohepatitis (NASH) mouse model, liver-specific E4bp4 knockout (E4bp4-LKO) mice present a lower degree of lipid droplet accumulation in the liver and a better liver function compared with those of E4bp4^flox/flox^ mice [34].

A latest study has elucidated the key role of E4bp4 in regulating the expression and activity of Cyp3a11 in mice [32]. Cyp3a11 is significantly upregulated in E4bp4^−/−^ mice than that of wild-type mice. Moreover, E4bp4 overexpression significantly downregulates, whereas its knockdown upregulates the mRNA and protein levels of Cyp3a11 in Hepa-1c1c7 cells in vitro [32]. As an upstream transcription factor, E4bp4 negatively regulates the expression level of Cyp3a11 both in vivo and in vitro. Their clinical significances in lipid metabolism in the liver, however, require a thorough exploration in the future.

During the process of weight fluctuation in obese mice, E4bp4 was significantly upregulated in WG group compared to NC group, but significantly downregulated in WL and WR groups compared to WG group. We also validated the opposite change in the expression level of E4bp4 to that of Cyp3a11 in the mouse liver. Therefore, we speculate that the E4bp4-Cyp3a11 axis is closely linked with weight fluctuation in obese mice.

Taken together, HFD induced weight gain, glucose and lipid metabolism disorders in mice, all of which were alleviated after feeding ND. Obese mice experienced weight regain after weight loss by re-feeding HFD, but their glucose and lipid metabolism disorders were milder than those induced by persistent obesity. The liver transcriptomic profile varied between mice with weight regain after weight loss and those with persistent obesity. During the process of weight fluctuation, E4bp4 and Cyp3a11 undergone simultaneous changes in the mouse liver. The E4bp4-Cyp3a11 axis may responsible for alleviating lipid droplet accumulation, weight loss and preventing weight rebound via mediating lipid metabolism in the liver. In the future, our findings should be validated by counterfactual experiments and a long-term observation. The underlying mechanism of the E4bp4-Cyp3a11 axis in regulating lipid metabolism and weight management requires a thorough analysis as well.

## Data Availability

The data that support the findings of this study are not openly available due to reasons of sensitivity and are available from the corresponding author upon reasonable request.
