# Correction: E4bp4-Cyp3a11 axis in high-fat diet-induced obese mice with weight fluctuation

**DOI:** 10.1186/s12986-024-00810-2

**Published:** 2024-07-19

**Authors:** Shuoshuo Sun, Ruixiang Zhang, Yu Chen, Yijiao Xu, Xingjia Li, Chao Liu, Guofang Chen, Xiao Wei

**Affiliations:** 1 Department of Endocrinology, Affiliated Hospital of Integrated Chinese and Western Medicine, Nanjing University of Chinese Medicine, Nanjing, 210028 People’s Republic of China; 2 Jiangsu Province Academy of Traditional Chinese Medicine, Nanjing, 210028 People’s Republic of China; 3 The Second Affiliated Hospital of Wenzhou Medical University, Wenzhou, 325000 People’s Republic of China

**Correction** **:** **Nutr Metab 21:30 (2024)**

**https://doi.org/10.1186/s12986-024-00803-1**

Following publication of the original article [1], the authors identified an error in Fig. 3. The correct figure is given below.


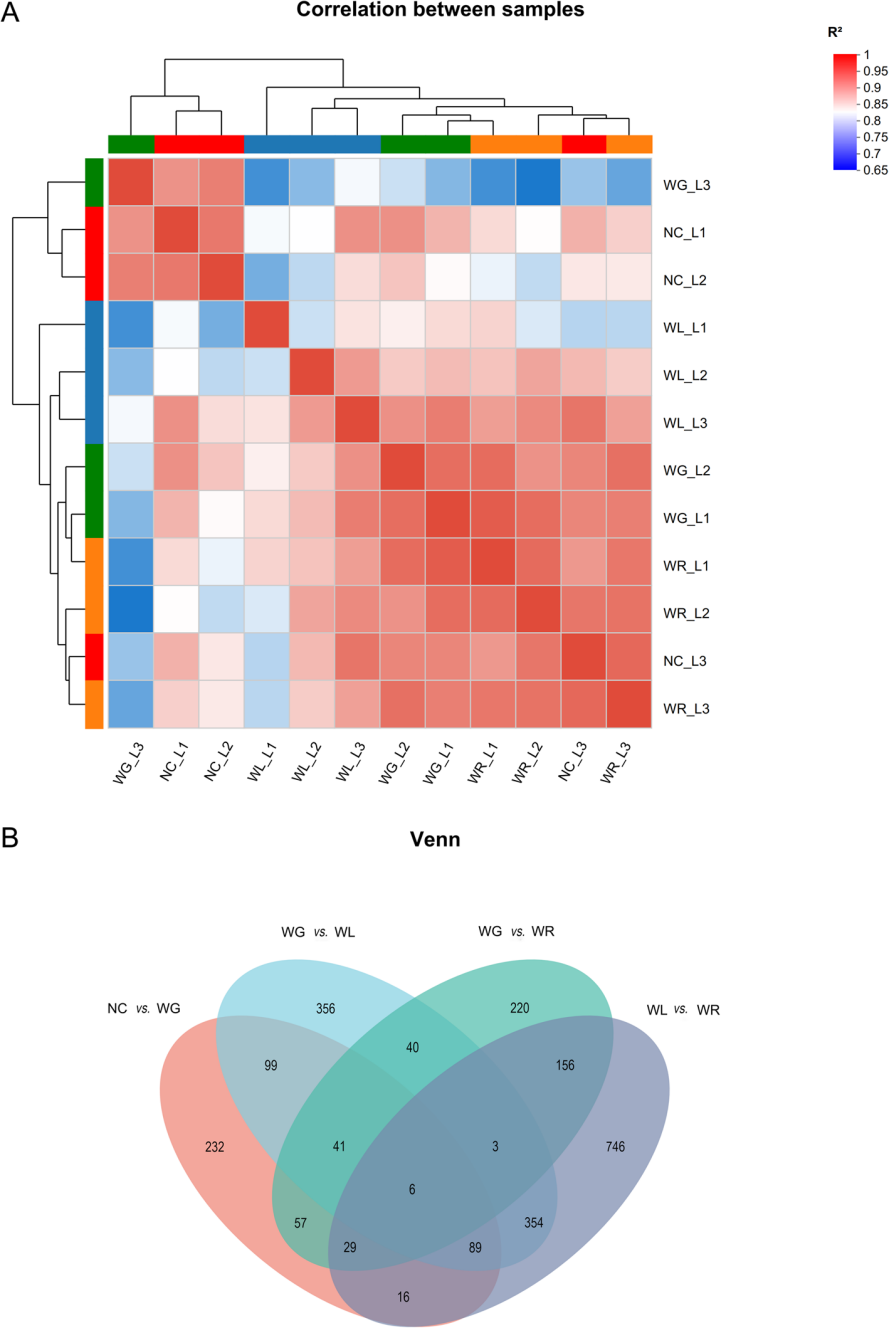


The original article [1] has been corrected.

## References

[1] Sun, et al. E4bp4-Cyp3a11 axis in high-fat diet-induced obese mice with weight fluctuation. Nutr Metab. 2024;21:30. 10.1186/s12986-024-00803-1. 10.1186/s12986-024-00803-1 PMC11131204 38802929

